# OCT1-target neural gene *PFN2* promotes tumor growth in androgen receptor-negative prostate cancer

**DOI:** 10.1038/s41598-022-10099-x

**Published:** 2022-04-12

**Authors:** Daisuke Obinata, Daigo Funakoshi, Kenichi Takayama, Makoto Hara, Birunthi Niranjan, Linda Teng, Mitchell G. Lawrence, Renea A. Taylor, Gail P. Risbridger, Yutaka Suzuki, Satoru Takahashi, Satoshi Inoue

**Affiliations:** 1 Department of Urology, Nihon University School of Medicine, 30-1, Ooyaguchikamicho, Itabashi-ku, Tokyo, 173-8610 Japan; 2 Prostate Cancer Research Group, Monash Biomedicine Discovery Institute Cancer Program, Department of Anatomy and Developmental Biology, Monash University, Wellington Road, Clayton, VIC 3800 Australia; 3 Department of Systems Aging Science and Medicine, Tokyo Metropolitan Institute of Gerontology, 35-2 Sakae-cho, Itabashi-ku, Tokyo, 173-0015 Japan; 4 Division of Neurology, Department of Medicine, Nihon University School of Medicine, 30-1, Ooyaguchikamicho, Itabashi-ku, Tokyo, 173-8610 Japan; 5 Cancer Research Division, Peter MacCallum Cancer Centre, 305 Grattan Street, Parkville, VIC 3000 Australia; 6 Sir Peter MacCallum Department of Oncology, The University of Melbourne, 305 Grattan Street, Parkville, VIC 3010 Australia; 7 Melbourne Urological Research Alliance (MURAL), Monash Biomedicine Discovery Institute Cancer Program, Monash University, Wellington Road, Clayton, VIC 3800 Australia; 8 Cabrini Institute, Cabrini Health, 183 Wattletree Road, Malvern, VIC 3144 Australia; 9 Prostate Cancer Research Group, Monash Biomedicine Discovery Institute Cancer Program, Department of Physiology, Monash University, Wellington Road, Clayton, VIC 3800 Australia; 10 Department of Computational Biology and Medical Sciences Graduate School of Frontier Sciences, University of Tokyo, 5-1-5, Kashiwanoha, Chiba, Chiba 277-8562 Japan; 11 Research Center for Genomic Medicine, Saitama Medical University, 1397-1 Yamane, Hidaka, Saitama 350-1241 Japan

**Keywords:** Oncogenes, Urological cancer

## Abstract

Androgen and androgen receptor (AR) targeted therapies are the main treatment for most prostate cancer (PC) patients. Although AR signaling inhibitors are effective, tumors can evade this treatment by transforming to an AR-negative PC via lineage plasticity. OCT1 is a transcription factor interacting with the AR to enhance signaling pathways involved in PC progression, but its role in the emergence of the AR-negative PC is unknown. We performed chromatin immunoprecipitation sequencing (ChIP-seq) in patient-derived castration-resistant AR-negative PC cells to identify genes that are regulated by OCT1. Interestingly, a group of genes associated with neural precursor cell proliferation was significantly enriched. Then, we focused on neural genes *STNB1* and *PFN2* as OCT1-targets among them. Immunohistochemistry revealed that both STNB1 and PFN2 are highly expressed in human AR-negative PC tissues. Knockdown of *SNTB1* and *PFN2* by siRNAs significantly inhibited migration of AR-negative PC cells. Notably, knockdown of *PFN2* showed a marked inhibitory effect on tumor growth in vivo. Thus, we identified OCT1-target genes in AR-negative PC using a patient-derived model, clinicopathologial analysis and an animal model.

## Introduction

Although androgen deprivation therapy (ADT) is initially effective for the treatment of prostate cancer (PC), inevitably castration-resistant tumors emerge^1^. Since androgen receptor (AR) signaling persists in most cases of castration-resistant PC (CRPC), second-generation AR signaling inhibitors are commonly used to treat CRPC^2–6^. However, with increasing use of AR signaling inhibitors, new mechanisms of resistance have emerged, including loss of AR expression, via lineage plasticity^7^.

AR signaling involves co-factors and AR-collaborating transcription factors (TFs)^8^. AR-collaborating TFs act on the AR-binding region condensed and packed by nucleosomes to support AR-binding, resulting in the enhanced expression of androgen-responsive genes. Among these TFs, GATA binding protein 2 (GATA2), forkhead box A1 (FOXA1), and octamer transcription factor (OCT1) were known to be significantly enriched along with functional AR in 68% of AR-binding regions^9^.

We previously demonstrated that high expression of OCT1 is correlated with poor outcome of PC patients^10^. Comprehensive analysis of OCT1 signals revealed that acyl-CoA synthase long-chain family member 3 (*ACSL3*) is the most highly expressed gene regulated by AR and OCT1 in hormone-responsive PC, LNCaP cells^11,12^. Moreover, we observed increased expression of OCT1 in CRPC tissues compared with hormone-sensitive PC^13^. The genome-wide analysis of OCT1-target genes in 22Rv1 cells, a model of AR-positive CRPC, identified another set of target genes distinct from LNCaP cells that accelerate PC cell proliferation by promoting cell cycle, including aniline-actin binding protein (*ANLN*) and DLG-associated protein 5 (*DLGAP5*)^13,14^. These data raise the hypothesis that the set of genes targeted by the OCT1/AR complex changes with PC progression.

On the other hand, AR-negative PC with neuroendocrine characteristics (NEPC) is initially a rare histological type that accounts for approximately 1% of PC patients, but it has highly malignant features^15^. However, the selective pressure of long-term androgen deprivation therapy (ADT) can eventually induce the development of NEPC^16^ or double (AR and NEPC marker) negative PC (DNPC). They arise in approximately 36% of patients treated with AR inhibitors^17^. Yet, the role of OCT1 in the transition of tumors to an AR-negative phenotype is still unknown.

Although PC3 and DU145 recapitulate AR-negative CRPC, they do not encompass the heterogeneity of CRPC. To address this challenge, efforts are underway to develop new in vivo, ex vivo, and in vitro models from samples of patients who have been treated. This includes patient-derived xenografts (PDX) which typically retain the histological and genome characteristics of the original patient’s tumor^18^. We have previously established new PDXs of CRPC that were resistant to conventional ADT, second-generation AR-directed inhibitors, and chemotherapy, one of which was an AR-negative CRPC model^19^. In the present study, we used this PDX model to elucidate the genome-wide network of OCT1 in AR-negative CRPC.

## Results

### Identification of OCT1-binding sites in AR-negative CRPC using ChIP-seq

**Figure 1:**
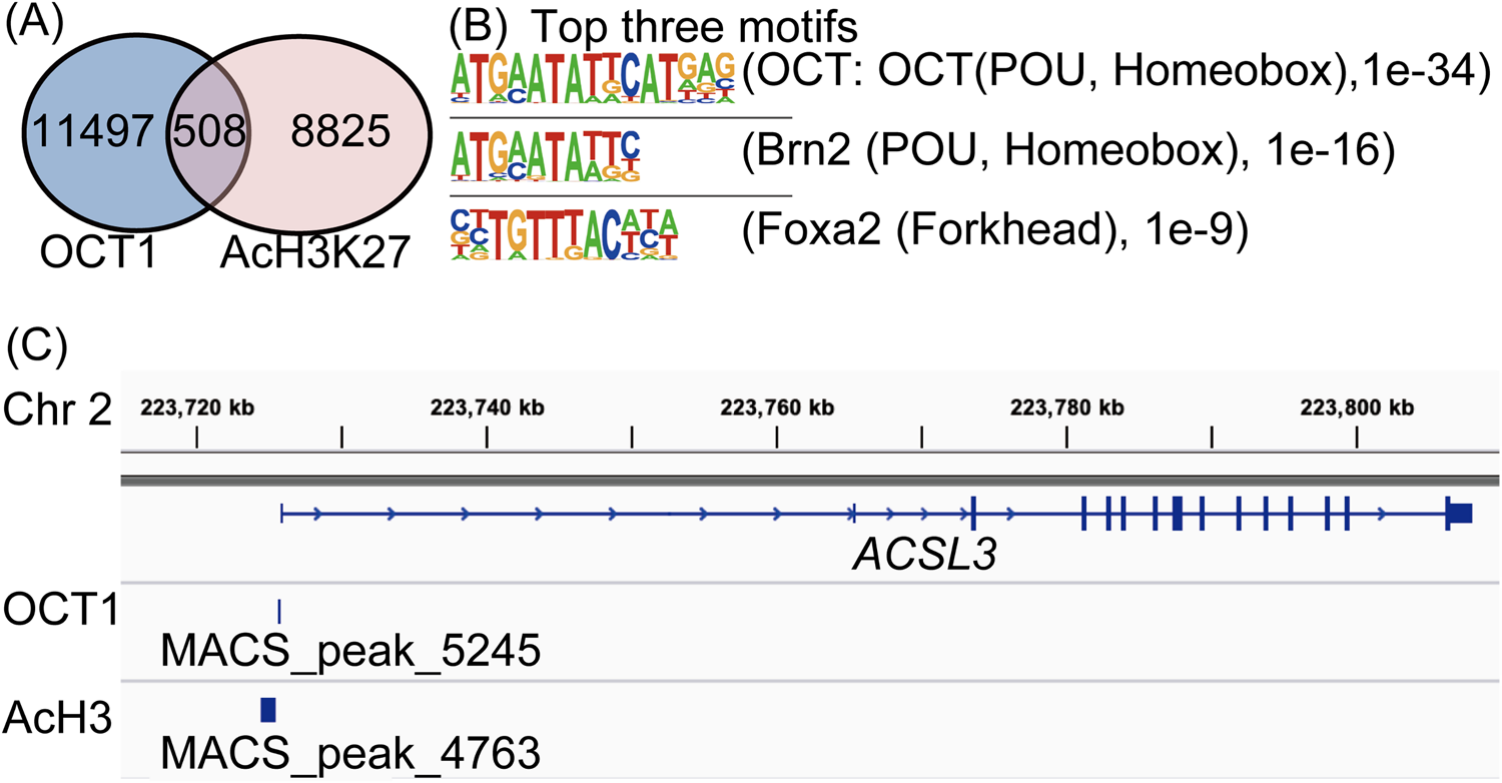
Global analysis of octamer transcription factor (OCT1) binding in androgen receptor (AR)-negative castration-resistant prostate cancer (CRPC) patient-derived xenograft (PDX). (**A**) Identification of Acetyl-Histone H3 (Lys27) (AcH3K27) and octamer transcription factor (OCT1)-binding regions by chromatin immunoprecipitation-sequencing (ChIP-seq). ChIP-seq analyses were performed using organoid established from PDX-201.2A-cx. AcH3K27and OCT1-binding regions (vs. input control, P < 1.0e−4) were determined by model-based analysis for ChIP-seq (MACS). (**B**) Motif analysis of OCT1-binding regions showing the enrichment of POU, Homeobox-binding motifs. We used HOMER motif analysis for 200-bp DNA sequences around OCT1-binding peaks. The two of top three motifs by this analysis are related to POU, Homeobox binding sequences. Octamer-Binding Protein 7 (Brn2), Forkhead Box A2 (Foxa1). (**C**) An outline of OCT1-binding regions in the vicinity of the representative OCT1-regulated gene acyl-CoA synthase long-chain family member 3 (ACSL3) on chromosome 2. AcH3K27 (AcH3), chromosome 2 (Chr 2).

We previously reported the distribution of OCT1-binding regions throughout the human genome in AR-positive PC cell lines, LNCaP, VCaP and 22Rv1^11,13^. We demonstrated that *OCT1* recruitment around AR binding sites (ARBS) is enhanced by androgens, and *OCT1*-regulated genes differ between castration-sensitive PC and AR-positive CRPC cells^11,13^. However, the role of OCT1 in AR-negative CRPC is unknown; hence we conducted ChIP-seq analysis to identify genome wide activated histone marker, histone H3 lysine (K) 27 acetylated (AcH3K27) sites, and OCT1-binding regions in PDX 201.2A-Cx [Gene Expression Omnibus (GEO) repository (www.ncbi.nlm.nih.gov/geo), accession number GSE193073]. PDX 201.2A-Cx is a patient-derived model of AR-negative CRPC that was established from a rapid autopsy sample of a lung metastasis from a PC patient who failed treatment with AR signaling inhibitors^19^. OCT1-binding regions were detected by calculating the enrichment of OCT1 compared with the input using the MACS software. In total, 12,005 binding regions were identified as OCT1 enriched sites and 9333 regions were identified as AcH3K27 enriched sites (vs. input control, P < 1.0e−4) (Fig. 1A). Among these, 508 regions overlapped (4.2% of OCT1 sites) (Fig. 1A). The HOMER program showed that the top two motifs are related to OCT family-binding motifs, suggesting direct OCT1 binding to the genome sequences with OCT family-binding motifs (Fig. 1B). Notably, Octamer-Binding Protein 7 (Brn2) is a major driver of neuroendocrine differentiation in prostate cancer^20^. We previously confirmed by luciferase assay that the enhancer region of *ACSL3* is indeed regulated by AR and OCT1 in LNCaP^11^, while only exhibited signal enrichments in the promoter region in PDX 201.2A-Cx cells, unlike cells from a model of AR-positive CRPC, where both *ACSL3* enhancer and promoter regions show signal enrichments (Fig. 1C)^13^. Using this ChIP-seq data and our previous data (GSE123565, GSE146886), we observed reduced histone acetylation and Oct1 bindings in promoter/enhancer regions of *AR* and other Oct1/AR target genes in the AR negative CRPC model (201.2A-Cx) compared with AR positive 22Rv1 cells (Supplementary Fig. S1). In contrast, high acetylation was observed in the genomic region of a NEPC marker, *SRY-Box Transcription Factor 2 (SOX2)*^21^, suggesting the changed transcriptional program and Oct1 target in the transition of AR positive state to AR negative.

### Combined analysis of hyperacetylated chromatin domains with OCT1-binding sites and RNA-seq data identified putative OCT1-regulated genes in AR-negative CRPC

**Figure 2:**
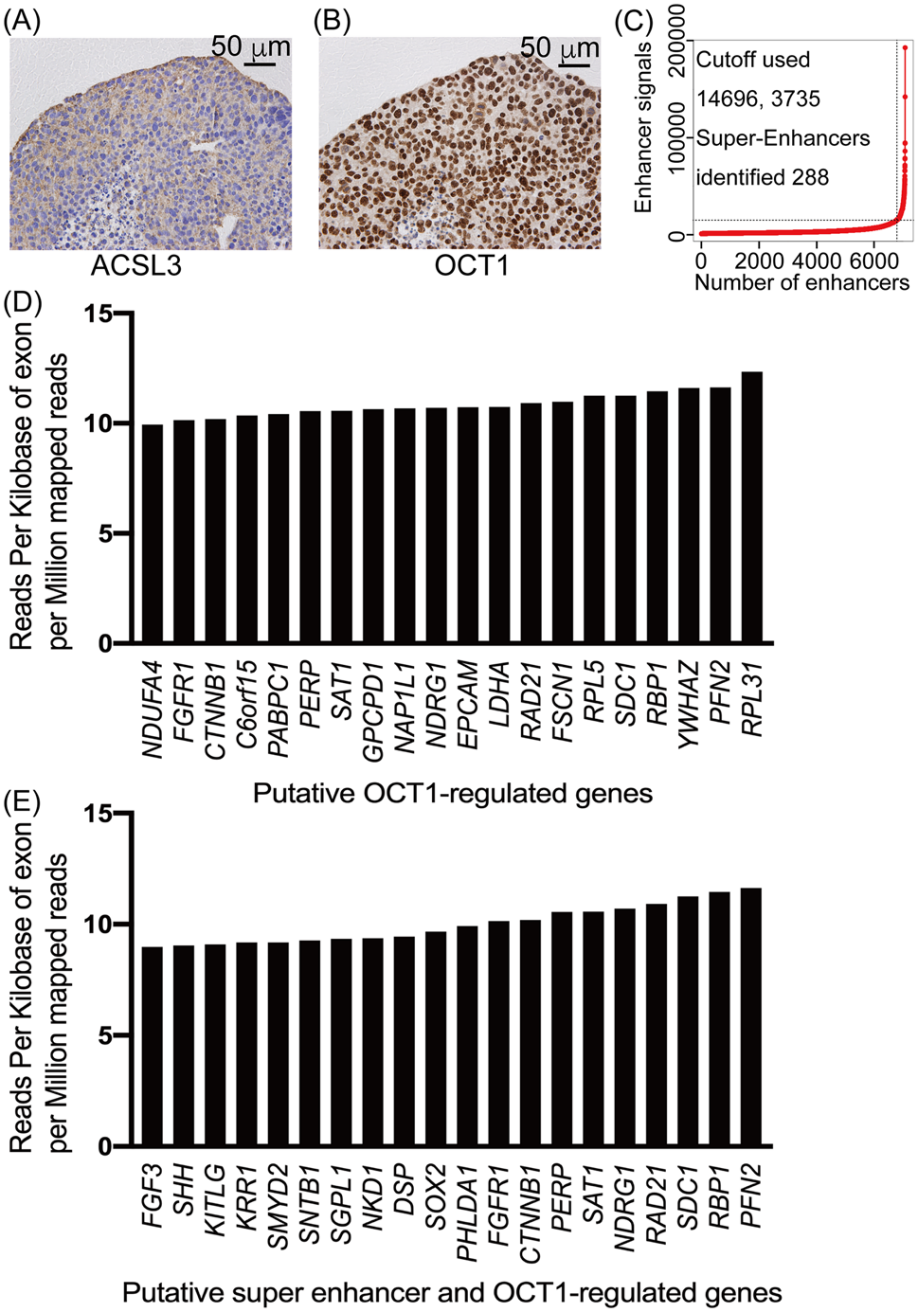
Analysis of OCT1 and putative OCT1-regulated gene expression in AR-negative CRPC. Representative images of immunohistochemistry for ACSL3 (**A**) and OCT1 (**B**) in PDX 201.2A-Cx. Scale bars equal 50 µm. (**C**) Line graph showing the number of putative super-enhancers defined by ranked AcH3K27 signal. (**D**) Top twenty highly expressed genes in the vicinity of OCT1-binding sites in PDX 201.2A-Cx. (**E**) Top twenty highly expressed genes among the putative super-enhancer associated genes in the vicinity of Oct1-binding sites in PDX 201.2A-Cx.

Next, we examined ACSL3 and OCT1 expression in the PDX 201.2A-Cx model of AR-negative CRPC using immunohistochemistry. OCT1 was strongly expressed, while ACSL3 was weakly expressed (Fig. 2A,B). We then investigated highly expressed OCT1-target genes by AcH3K27 ChIP-seq data in 201.1A-Cx. In addition, Rank Ordering of Super-Enhancers (ROSE) analysis^22^ revealed enhancers and putative super-enhancers using ChIP-seq signals for AcH3K27 in PDX 201.2A-Cx cells, and identified 288 putative super-enhancers (Fig. 2C). Furthermore, using Genomic Regions Enrichment of Annotations Tool (GREAT) version 4.0.4, we detected 669 putative OCT1-regulated genes located within 1000 kb of the OCT1 binding signal overlapping with significant AcH3K27 sites. Among them, 168 genes were identified to be putative super-enhancers and OCT1-regulated genes, which overlapped with the putative super-enhancer regions.

We used our previous RNA-seq data^19^, to examine the mRNA abundance of these genes. The top 20 genes based on high expression levels are shown for putative OCT1-target genes (Fig. 2D) and putative super-enhancer and OCT1-regulated genes (Fig. 2E). Several genes were present in both categories, including Catenin Beta 1 (*CTNNB1),* Fibroblast Growth Factor Receptor 1 (*FGFR1),* N-Myc Downstream Regulated 1 *(NDRG1),* P53 Apoptosis Effector Related To PMP22 *(PERP),* profilin 2 (*PFN2*)*,* RAD21 Cohesin Complex Component *(RAD21),* Retinol Binding Protein 1 *(RBP1),* Spermidine/Spermine N1-Acetyltransferase 1 *(SAT1),* and Syndecan 1 *(SDC1)*.

### Identification of OCT1-regulated genes related to neural function

**Figure 3:**
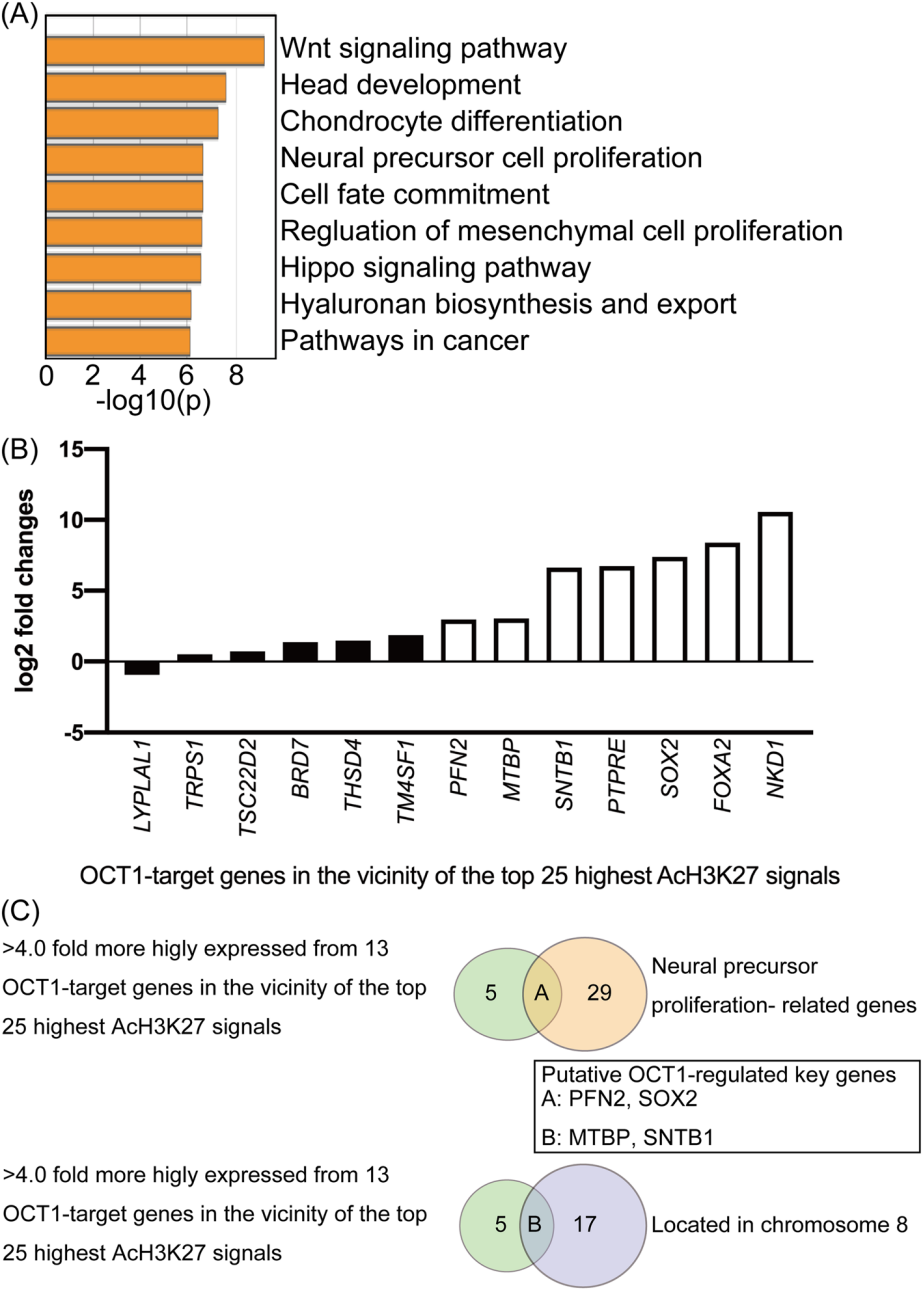
Identification of putative OCT1-regulated genes. (**A**) Functional annotations for putative super-enhancer associated OCT1 putative target genes in PDX 201.2A-Cx. (**B**) The log2-fold changes against AR-positive CRPC tissues in gene expression of 13 OCT1 target genes in the vicinity of the top 25 highest AcH3K27 signals of the top 100 most highly expressed genes in AR-negative CRPC tissues. (**C**) Venn diagram showing unique and common features of putative super-enhancer-associated genes in the vicinity of OCT1-binding sites.

To evaluate the relationship between OCT1 binding and gene function, we performed functional gene set analysis using Metascape which is a web-based analysis tool that provides a comprehensive gene list annotation and analysis resource^23^. We extracted a list of the top 100 most highly expressed genes among putative super-enhancer and OCT1-regulated genes. We found that these genes are associated with various annotation groups and are significantly enriched in the Wnt signaling pathway, head development, chondrocyte differentiation, and neural precursor cell proliferation (Fig. 3A). Of note, it is interesting to find that a group of neural precursor cell proliferation, which may influence NEPC proliferation, were enriched.

To prioritize putative OCT1 target genes for further analysis, we used a filtered the dataset using several criteria. First, we focused on chromosome 8, which is commonly amplified in prostate cancer^24^. These amplifications include MYC, which promotes prostate tumorigenesis^25^ and has a copy number gain in 201.2-Cx^19^.

In addition, we selected 13 OCT1-target genes in the vicinity of the top 25 putative super-enhancer regions among the top 100 most highly expressed putative super-enhancer and OCT1-regulated genes (Fig. 3B). We also examined which genes were more highly expressed in the AR negative versus AR positive PDX from the same patient using our previously reported RNA-seq data^19^ (Fig. 3B). We found seven putative super-enhancer and OCT1-regulated genes were more highly (> 4.0 fold) expressed in AR negative CRPC than in AR-positive CRPC tissues.

**Figure 4:**
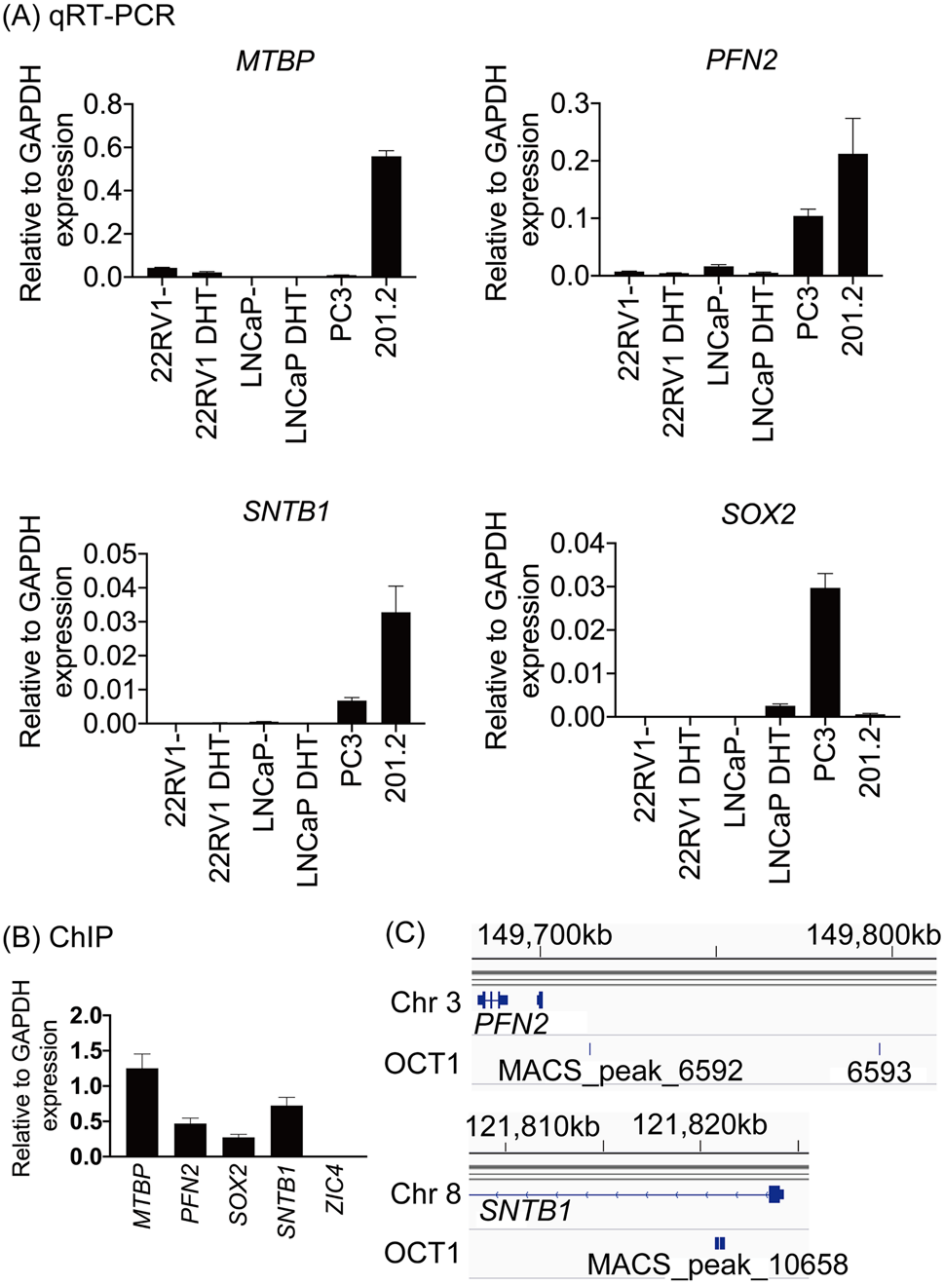
The expression characteristics of candidate OCT1-regulated genes. (**A**) RNA expression levels of each candidate OCT1 regulated gene in representative prostate cancer cell lines and organoids grown from PDX 201.2A-Cx (201.2). 100 nM of dihydrotestosterone treatment (DHT), Bars, standard error of the mean (SEM). (**B**) ChIP analysis of OCT1 binding in the enhancer regions of each gene in 201.2A. The regulatory region of Zic Family Member 4 *(ZIC4)* was used as negative control. The OCT1 binding region targeted by quantitative reverse transcription PCR (qRT-PCR) and the corresponding gene are shown below. MDM2 Binding Protein (*MTBP*): model-based analysis for ChIP-seq (MACS)_peak_10653, profilin 2 (*PFN2*): MACS_peak_6593, SRY-Box Transcription Factor 2 (*SOX2*): MACS_peak_6774, and Syntrophin Beta 1 (*SNTB1*): MACS_peak_10658. Bars, standard deviation (SD). (**C**) Images of OCT1-binding regions in the vicinity of the putative OCT1-regulated genes *PFN2* and *SNTB1*.

Next, we identified two genes that are related to neural precursor cell proliferation. In addition, two genes are identified to be located in chromosome 8 (Fig. 3C). Therefore, we focused on these four genes, *profillin2* (*PFN2)*, *SOX2*, Syntrophin Beta 1 (*SNTB1*), and MDM2 Binding Protein (*MTBP*). To validate the expression level of these genes, we performed qRT-PCR using AR-positive and -negative PC cell lines as well as PDX 201.2A-Cx cells. All genes were more highly expressed in PDX 201.2A-Cx cells than the cell lines, except for *SOX2* (Fig. 4A). Moreover, we performed ChIP analysis with the OCT1 antibody to validate OCT1 binding. We observed significant enrichment of OCT1-binding in the regulatory regions of all genes, but not the regulatory region of Zic Family Member 4 (*ZIC4)*, which was used as negative control (Fig. 4B,C).

### SNTB1 and PFN2 are positively regulated by OCT1 and highly expressed in bone metastatic prostate cancer tissues

**Figure 5:**
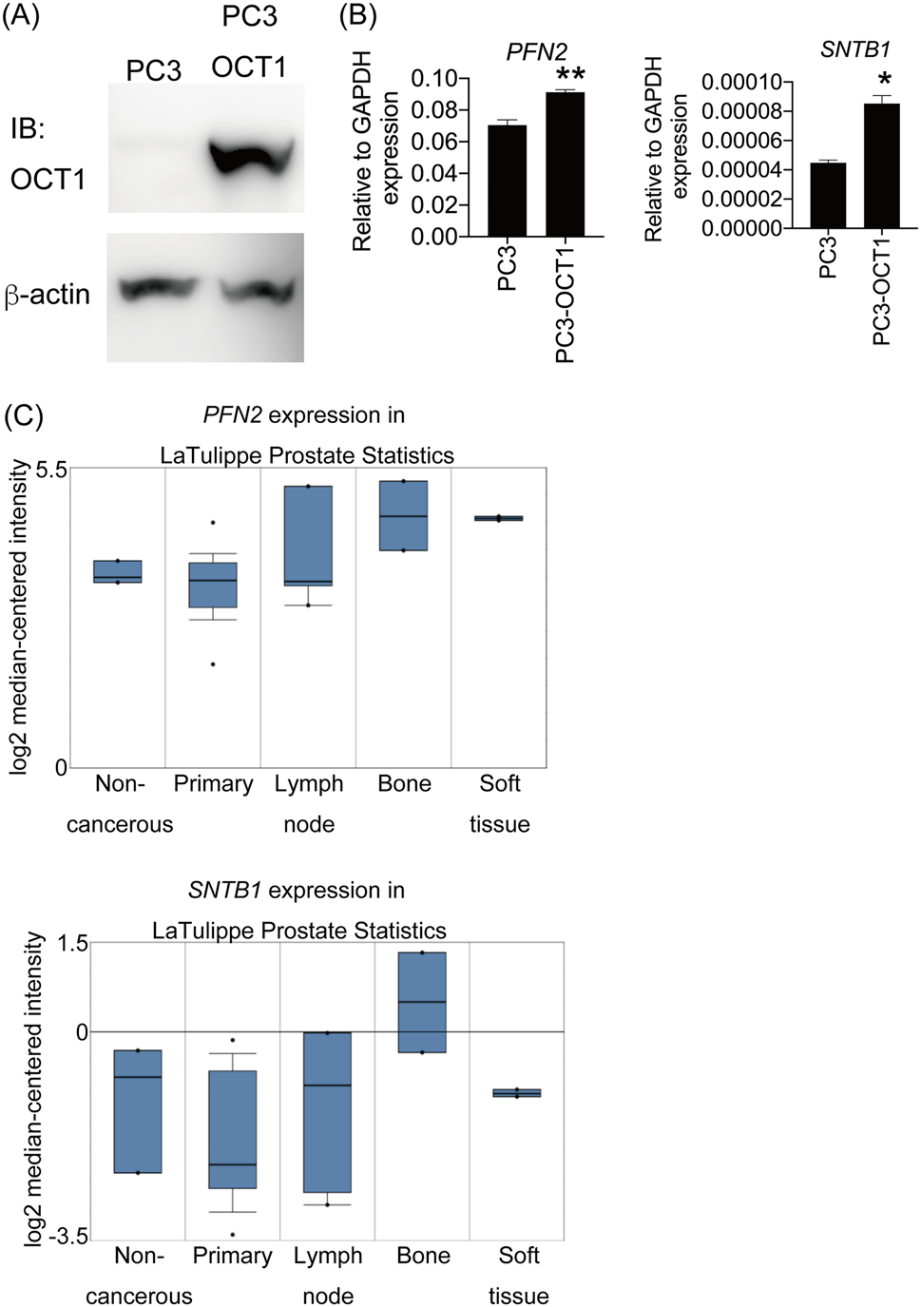
OCT1 positively regulates PFN2 and SNTB1. The effect of transient transfection of OCT1 on gene expression. PC3 cells were transfected with or without OCT1 expression plasmid (PC3-OCT1 and PC3) for 48 h. OCT1 expression was analyzed by Western blotting (**A**), and expression of each candidate gene was analyzed by qRT-PCR. *p < 0.05, **p < 0.01, Student’s t-tests. Bars, SEM (**B**). (**C**) Expression of *SNTB1* and *PFN2* mRNA is higher in bone metastatic prostate cancer tissues. These expression levels were analyzed using a public database in Oncomine^49^. The original Western blots are presented in Supplementary Fig. S2.

To investigate the relationship between the putative OCT1-target genes and OCT1, we performed transient transfection of OCT1 into PC3 AR-negative PC cells (Fig. 5A and Supplementary Fig. S2). Increased expression of *SNTB1* and *PFN2* was detected in both OCT1 transfected cells, whilst *MTBP* was decreased (Fig. 5B and Supplementary Fig. S3). Thus, we analyzed *SNTB1* and *PFN2* gene expression in prostate adenocarcinoma tissues using Oncomine. Interestingly, we found that the expression levels of these two genes were higher in bone metastases than in primary tumors (Fig. 5C).

### Immunohistochemistry of PFN2 and SNTB1 in clinical CRPC tissues and inhibitory effects on migration and tumor growth by PFN2 knockdown

**Figure 6:**
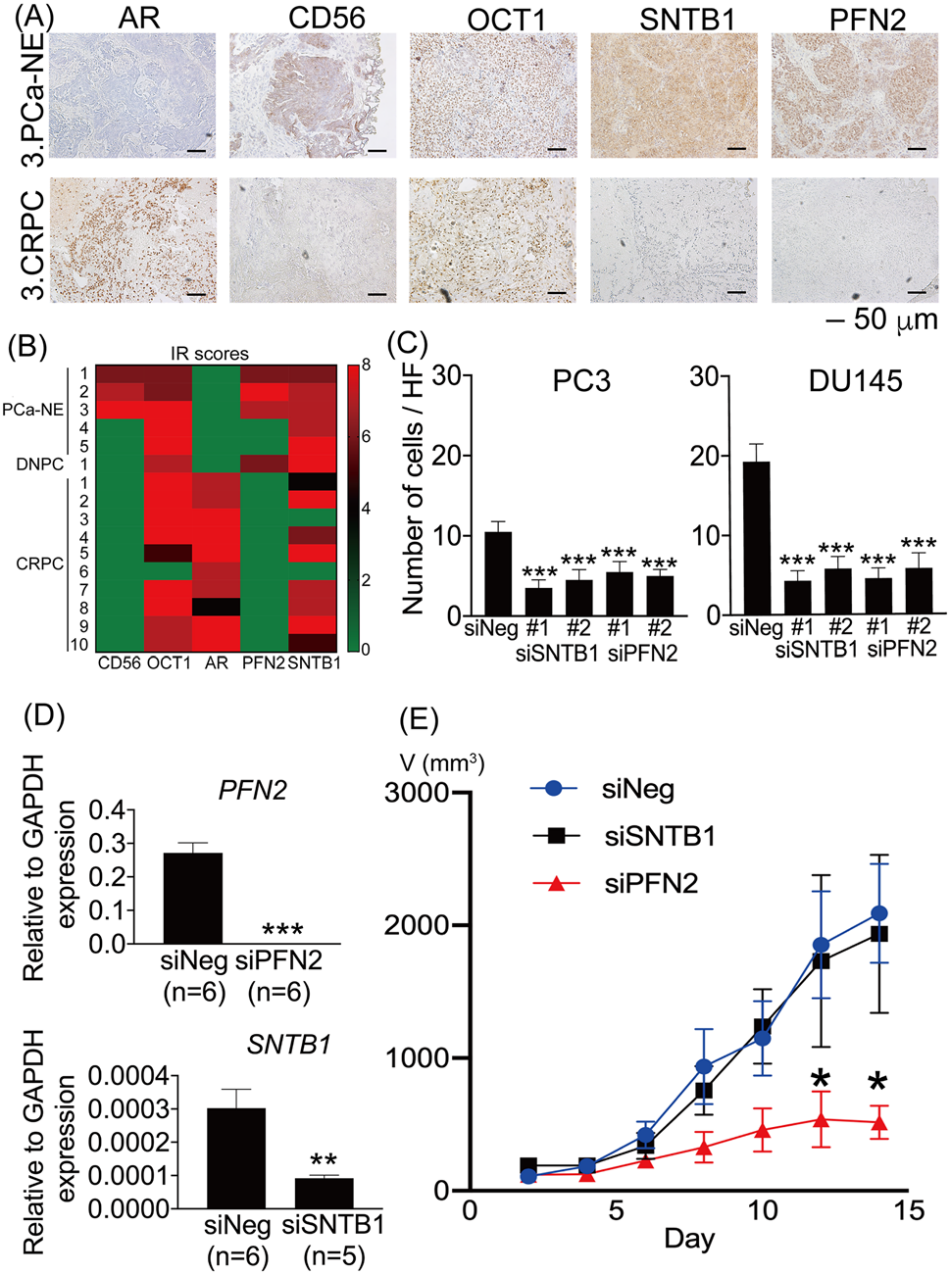
Immunohistochemistry of PFN2 and SNTB1 in clinical CRPC tissues and functional analysis in AR-negative CRPC model cells. (**A**) Representative images of immunohistochemistry for NEPC (PCa-NE; N = 5) and CRPC cases (N = 10). (**B**) Results of immunohistochemistry using clinical specimens. The expression level of each protein using the immunoreactivity (IR) score is summarized in the heat map. Note that, in the case of 2. bladder tumor (BT)/PCa-NE, the bladder and prostate were resected endoscopically, so it was unable to determine whether it originated from the prostate or the bladder. DNPC: AR and NE marker double negative PC (N = 1). (**C**) Data from cell migration assays where the number of migratory cells was counted in five random fields and compared. Both siRNAs inhibited the migration ability on PC3 and DU145. ***p < 0.001, One-way ANOVA with Dunnett’s multiple comparisons test. Bars, SD. siNegative Control (siNeg), siRNA targeting *SNTB1* (siSNTB1), siRNAs targeting *PFN2* (siPFN2). (**D**) RNA was extracted from harvested tumors and qRT-PCR was performed. ***p < 0.001 for siPFN2 vs. siNeg, **p < 0.01 for siSNTB1 vs. siNeg, Student’s t-tests (n = 6 each). Bars, SEM. (**E**) siPFN2 significantly reduced the volume of tumors compared to the volume when siNeg was administered. On the other hand, siSNTB1 did not show any significant difference. The mean volume (V mm^3^) of tumors formed in mice was shown and calculated by the following formula: V = 0.5 × maximum diameter × intermediate diameter × minimum diameter. *p < 0.05 for siPFN2 vs. siNeg, bars, SD, Student’s t-tests for each day (n = 6 each, one mouse in the SNTB1 group died during the course of the study). Bars, SD.

Since the expression of SNTB1 and PFN2 in AR-negative CRPC has not been reported, immunohistochemistry was performed on tissues from 16 cases of NEPC, DNPC or CRPC. Then, we revealed that SNTB1 and PFN2 proteins were positively expressed in most of AR-negative PC tissues including NEPC (Fig. 6A,B). To investigate the effects of altered expression of these two genes on the proliferation and migration of AR-negative CRPC cells, PC3 and DU145 were transfected with siRNA targeting *SNTB1* and *PFN2* (si*SNTB1* and si*PFN2*). We confirmed that these siRNAs significantly reduced the expression of endogenous *SNTB1* and *PFN2* in DU145 and PC3 cells (Supplementary Fig. S4). PC3 and DU145 cells transfected with si*SNTB1* and si*PFN2* showed no obvious change in proliferation compared to those transfected with siNegative Control. However, there was a significant decrease in migration in both cell lines (Fig. 6C and Supplementary Fig. S5). Meanwhile, we showed that transient expression of OCT1 increased the migration of PC3 cells (Supplementary Fig. S6).

To explore in vivo roles of these genes, xenografts of PC3 cells in male nude mice were injected with si*PFN2*, si*SNTB1*, or siNegative Control. Similar to the in vitro results, qRT-PCR and immunohistochemistry of harvested tumor specimens showed that both *PFN2* and *SNTB1* expression levels in the tumors were reduced by each siRNA (Fig. 6D, Supplementary Fig. S7A,B). Notably, whereas the si*SNTB1*-treated tumors had a similar growth rate to those treated with siNegative Control, there was a significant reduction in tumor volume in tumors treated with siPFN2 (Fig. 6E). Visceral metastases or lymph node swelling were not observed. Using GEPIA, an online tool for analyzing the RNA-seq expression data^26^, *PFN2* high expression is significantly correlated with poor overall survival of prostate cancer patients in The Cancer Genome Atlas (TCGA) dataset (Supplementary Fig. S8), while *SNTB1* is not.

## Discussion

The AR plays an important role in prostate cancer, even in the development of CRPC. Although ASRI are used after or in combination with androgen deprivation therapy (ADT), AR-positive PC cells can adapt into AR-negative phenotype, becoming independent of AR expression or signaling^7^. Furthermore, CRPC is a highly heterogeneous disease unlike conventional cell lines. Thus, the lack of in vitro prostate cancer models that recapitulate the diversity of human prostate cancer has prevented an understanding of pathogenesis and treatment response of the disease. We previously focused on four PDXs, one of which was AR-negative CRPC, from rapid autopsy samples of two CRPC patients who had exhausted multiple therapies, and confirmed that they recapitulate the phenotypic diversity of CRPC by genomic profiling^19^. We also examined the core pathways enriched throughout these PDXs based on RNA-seq data^19^. To understand the role of core transcription factors in AR-negative CRPC, we profiled OCT1 binding in a patient-derived model and identified two OCT1-target genes that promote the migration and/or tumor growth of AR-negative PC.

The distribution of AR-binding regions as well as the type of TFs cooperating with AR are different between hormone-sensitive and castration-resistant PCs. Although the CRPC-specific AR-binding regions are not occupied with typical AR-collaborating factors as observed in hormone-sensitive PC cells, the AR independent TFs, such as MYC, are recruited into these sites^27^. These data suggest that the type of TFs recruited into the AR-binding region change in accordance with lineage plasticity. On the other hand, OCT1 is widely expressed, including in the placenta, rectum and testis (Human Protein Atlas available from http://www.proteinatlas.org), in the human body among the eight OCT proteins, and known to be associated with the master regulator of pluripotency, octamer-binding transcription factor 4 (OCT4), in stem cells^28–30^. We recently reported that OCT4 associates with Nuclear Respiratory Factor 1 (NRF1) in AR-negative PC and consists an important component of the TF complex in CRPC^31^. Interestingly, OCT1 binding sites were enriched in a series of genes that are regulated by MYC in MYC-driven lung adenocarcinomas^32^, suggesting that OCT1 and MYC may co-regulate a series of androgen-responsive genes in PC. Consistent with these results, our PDXs are shown to express high levels of MYC^19^. We have previously reported that OCT1-regulated genes that overlap with AR signals differ between LNCaP and 22Rv1 cells^11,13,14^. Here, using a PDX model of AR-negative PC, we demonstrated the enrichment of OCT1-bound genes associated with proliferation of neural progenitor cells, which may be independent of the AR network. Interestingly, some of the genes we focused on in this study were mildly repressed in an androgen-dependent manner in LNCaP and 22Rv1 (Fig. 4A). Genes involved in cellular plasticity, such as MYC and SOX2, are known to be negatively correlated with AR^33^. Androgen deprivation can induce the expression level of androgen-repressed genes, leading to high expression in CRPC^34,35^. Therefore, we expect that negative regulation of these genes might be correlated with high expression level in our PDX. Loss of AR expression in this models is not due to genomic loss, but likely due to epigenetic changes^19^. Accordingly, there was no significant histone H3K27 acetylation in the *AR* promoter region in this PDX model (Supplementary Fig. S1). Although further analysis of the epigenome is required, such as DNA methylation, we hypothesize that epigenetic changes in this locus may affect the distribution of TFs, including OCT1, during the phenotypic transition of prostate cancer cells. Thus, these results suggest that in addition to the types of AR collaborating TFs changing with disease progression, the genes regulated by the TFs also change significantly.

Concerning the two OCT1-target genes in AR-negative PC, *PFN2* and *SNTB1*, that were focused on in this study, the functions of SNTB1 are not well characterized. PDX-201.1A-Cx was generated from a metastasis, and Oncomine data shows that these genes are more highly expressed in metastases than in primary tumors. SNTB1 has been associated with intense myopia and muscle contraction^36,37^. On the other hand, PFN2, a member of profilin family, regulates actin polymerization in response to extracellular signals by binding to an actin monomer and is essential for controlling cytoskeletal movement, particularly in synapses where it regulates vesicle release, neuronal excitability, and ultimately complex behavior^38^. Recent reports have shown that aberrant expression of *PFN2* is associated with poor prognosis in breast cancer and that *PFN2* is regulated by *FOXD2-AS1* and *OCT4*^39^*.* Furthermore, *PFN2* was shown to be upregulated in head and neck cancers and its knockdown inhibited proliferation, invasion and migration of these cells. It is supposed that PFN2 activates the PI3K/Akt/β-catenin signaling pathway in head and neck cancers^40^. Meanwhile, there has been no previous report on the relationship between *PFN2* and PC cells to our knowledge. In our study, siPFN2 and siSNTB1 both inhibited migration in vitro, but interestingly, only siPFN2 markedly inhibited tumor growth in vivo. This might be caused by the lower expression of *SNTB1* in PC3 cells compared to *PFN2*. It is tempting to speculate that *PFN2* is involved in epithelial mesenchymal transition, which may affect cell migration and tumorigenicity in vivo. Moreover, the mechanical properties of a cell are likely to be crucial for its migratory behavior. Previous reports have shown that spatial cytoskeletal organization is required for the deformation of cancer cells to promote migration^41^. Therefore, it is possible that PFN2 enhances migration by regulating actin polymerization for cytoskeleton organization.

In conclusion, this study uncovered global OCT1-binding sites in a patient-derived model of AR-negative PC. Neuronal cell proliferation associated signals were significantly enriched among OCT1-targets in this model. We identified OCT1-target genes in AR-negative PC cells, including *PFN2*, which affects the growth of AR-negative PC tumors. Although additional in vivo experiments and AR negative PDXs are necessary to validate the results of this study, we propose that targeting *PFN2* could be a promising therapeutic strategy for aggressive AR-negative phenotypes of CRPC.

## Materials and methods

### Cell culture and reagents

PDX 201.2A-Cx was provided by the Melbourne Urological Research Alliance (MURAL)^19^ and cultured as organoids. Briefly, PDX 201.2A-Cx was minced and digested in growth factor reduced, phenol red-free, ldEV-free Matrigel and cultured in advanced DMEM/F-12 media containing 1% penicillin–streptomycin, 2 mM Glutamax, 1 nM DHT, 1.25 mM *N-*acetylcysteine, 50 ng/mL EGF, 500 nM A83-01, 10 mM, nicotinamide, 10 μM SB202190, 2% B27, 100 ng/mL noggin, 10 ng/mL FGF10, 5 ng/mL FGF2, 1 μM prostaglandin E2 and 10% R-spondin 1 conditioned media. 10 μM Y-27632 dihydrochloride as previously described^19^. The PC cell lines LNCaP [Research Resource Identifier (RRID): CVCL_0395], 22RV1 (RRID: CVCL_1045), PC3 (RRID: CVCL_0035) and DU145 (RRID: CVCL_0105) were purchased from American Type Culture Collection (Rockville, MD, USA). LNCaP, 22RV1, PC3 are cultured using RPMI-1640 and DU145 using DMEM medium, respectively, and both are supplemented with 10% FBS, 50 U/mL penicillin and 50 μg/mL streptomycin, in addition, some of LNCaP and 22Rv1 cells are treated with 100 nM of dihydrotestosterone (DHT) (Wako, Tokyo, Japan) for 3 days^14^. Short tandem repeat (STR) analysis was performed to authenticate the cell lines used in the present study^31,42^. Also, these cells have been checked for Mycoplasma contamination using real-time PCR performed by Funakoshi Co., Ltd. (Tokyo, Japan).

### Chromatin immunoprecipitation-sequencing (ChIP-seq)

OCT1 and Acetyl-Histone H3 (Lys27) (AcH3K27) ChIP-seq with organoids from PDX 201.2A-Cx was performed as in previous studies using an Illumina HiSeq 2500 (Illumina, San Diego, CA, USA)^13^. The signal score for OCT1 binding was calculated using Model-based analysis of ChIP-seq (MACS; 20). The threshold for binding sites was set at P < 1.0e−4. The dataset was examined using the Integrative Genome Viewer. Genes located within 1000 kb of the upstream and downstream of the binding site were extracted with the Genomic Regions Enrichment of Annotations Tool (GREAT) version 4.0.4 to be candidates for OCT1-target genes^43^. Motif search (50 bp around the peaks obtained by ChIP-seq) was performed using HOMER^44^, and the samples used for ChIP-seq were further validated for binding to some regions by qPCR as described below. To identify putative super enhancers, we used Rank Ordering of Super-Enhancers (ROSE) downloaded from Young Lab (http://younglab.wi.mit.edu/super_enhancer_code.html)^22^.

### Quantitative reverse transcription PCR (qRT-PCR)

Total RNA extraction of all PC cell lines and PDX 201.2A-Cx, first-strand cDNA synthesis, and qRT-PCR were performed. Total RNA was obtained using RNeasy Mini kit (QIAGEN, Tokyo, Japan). First-strand cDNAs were generated using the PrimeScript RT reagent kit (Takara, Kyoto, Japan) according to the manufacture’s protocol. The expression level of genes was measured by qRT-PCR using the SYBR green mix (Applied Biosystems, Waltham, MA, USA)^45^. The expression level of each gene as a ratio to the expression level of glyceraldehyde-3-phosphate dehydrogenase (GAPDH) was calculated by the ΔΔCt method^46^. The primers used in this study are shown in the Supplementary Table S1. For the MYC primer, we referred to a previous report^47^.

### Transient expression of OCT1 in PC3 cells

We used an OCT1 pcDNA3.1-FLAG construct as previously reported^11^. PC3 cells were transfected with 1000 ng OCT1-pcDNA3.1-FLAG (PC3-OCT1) or without plasmids for control cells (PC3) using X-tremeGENE HP DNA transfection Reagent (Roche Applied Science, Basel, Switzerland) according to manufacturer’s protocol.

### Western blot analysis

PC3 and PC3-OCT1 were thawed on ice and whole protein lysates extracted from these cells using RIPA lysis buffer with protease inhibitor (nacalai tesque, Kyoto, Japan). Lysates were mixed with NuPAGE™ Sample Reducing Agent (10X) and NuPAGE™ LDS Sample Buffer (4X) (Thermo Fisher Scientific, Tokyo, Japan) and loaded on Bis–Tris Gels (Thermo Fisher Scientific), separated by electro-phoresis. Samples were transferred to Immobilion-P Transfer Membranes (Millipore, Billerica, MA, USA). Membranes were incubated with primary antibodies overnight and then were incubated with secondary antibodies. Chemiluminescence was achieved with Chemi-Lumi One Super (nakalai tesque). The following antibodies were used in this study: Rabbit polyclonal anti-OCT1 antibody (abcam, Cambridge, UK)^10^, Mouse monoclonal anti-β-actin monoclonal antibody (Sigma, St Louis, MO, USA)^10^.

### Immunohistochemistry

Immunohistochemistry was performed on PDX tissue using the Leica BOND-MAX-TM automated system (Autostainer) with BondTM epitope retrieval (ER)-1 for anti-ACSL3 Abnova (Taipei, Taiwan) and ER-2 for anti-OCT1 antibody^19^. Prostate cancer tissues obtained by prostate biopsy were stained by the streptavidin–biotin amplification method^48^. The use of prostate cancer tissue for immunohistochemistry has been approved by the Institutional Review Board and the Research Ethics Committee of the Nihon University School of Medicine (approval number: RK-190611-03). Briefly, anti-SNTB1 and anti-PFN2 polyclonal antibodies (HPA024659 and HPA35611 from Sigma, 1:200 dilution) were applied, followed by Histofine Simple Stain MAX-PO (Nichirei, Tokyo Japan). The antigen–antibody complex was visualized with 3,30-diaminobenzidine (DAB) solution (1 mM DAB, 50 mM Tris–HCl buffer [pH 7.6], and 0.006% H_2_O_2_). Immunoreactivity (IR) score (0–8) was obtained as the sum of the proportion and the intensity of immunoreactivity. Proportion (0, none; 1, < 1/100; 2, 1/100 to 1/10; 3, 1/10 to 1/3; 4, 1/3 to 2/3; and 5, > 2/3), Intensity (0, none; 1, weak; 2, moderate; and 3, strong).

### Small interfering RNA

We transfected cells with 10 nM of siRNA using Lipofectamine RNAi MAX (Thermo Fisher Scientific, Waltham, MA, USA) according to the manufacture’s protocol. We purchased Silencer Select Negative Control #1 siRNA (siNegative Control, #4390844) and Silencer Select siRNAs (#44427037) targeting *SNTB1* (siSNTB1, #1: s13252, #2: s13253) and *PFN2* (siPFN2, #1: s10378, #2: s10379) from ThermoFisher Japan (Tokyo, Japan).

### Migration assay

Migration assays were performed by using the Cell Culture Insert with 8.0-μm pore size polyester filters (BD Biosciences). We added 700 μL of cultured medium for PC3 or DU145 cells in the lower chamber and 300 μL of cultured medium containing cells transfected with siRNAs (5 × 10^3^) in the upper chamber. After 24 h of incubation, the cells on the lower side of inserts were fixed, then stained with Giemsa solution (Muto Pure Chemicals, Tokyo, Japan). The cells that migrated on the lower surface were counted in four randomly selected fields under a microscope at a magnification of × 40.

### Cell proliferation assay

PC3 and DU145 cells transfected with siRNA complex were seeded at 5000 cells/well in 96 well plates and cultured in RPMI-1640 and DMEM. At the indicated time points, cell proliferation was quantified using 2-(2-methoxy-4-nitrophenyl)-3-(4-nitrophenyl)-5-(2,4-disulfophenyl)-2H tetrazolium, monosodium salt (WST-8), Cell Count Reagent SF (Nacalai Tesque, Kyoto, Japan) according to the manufacturer’s instructions. Assays were performed in four wells and data were presented as average and SD.

### In vivo xenograft experiment

In vivo study was approved by the animal ethics committee of Nihon University School of Medicine (Approval number: AP18MED051-1). All animal experiments described adhere to policies and practices approved by the Institutional Animal Care and Use Committe (IACUC). The study was performed in compliance with the ARRIVE guidelines (2010). PC3 cells (3 × 10^6^ cells) were injected subcutaneously into the right flank of 5-week-old male nude mice (BALB/cA-nu/nu obtained from Oriental Yeast, Tokyo, Japan) (total n = 18). Tumor size was monitored by caliper measurements every 3 days, and the volume of tumors was calculated by the following formula: V = 0.5 × maximum diameter × intermediate diameter × minimum diameter. When tumor size reached approximately 100 mm^3^, 5 μg of siNegative control, siPFN2, or siSNTB1 (siPFN2: Silencer select Pre-Designed siRNA ID: s10378, siSNTB1: Silencer select Pre-Designed siRNA ID: s13252) mixed with RNAi MAX Transfection Reagent was injected into the tumor twice weekly for 2 weeks. Xenografts were harvested from mice anesthetized with isoflurane and stained with SNTB1 and PFN2 antibodies, and homogenized in ISOGEN (Nippon Gene, Tokyo, Japan) solution to extract RNA for cDNA.

### Analysis of clinical data in the public databases

We compared the transcriptional expression of *PFN2* and *SNTB1*, and prognosis of prostate cancer cases based on publicly available data from Gene Expression Profiling Interactive Analysis (GEPIA)^26^ and Oncomine databases (https://www.oncomine.org/)^49^. Using data from the Cancer Genome Atlas database, GEPIA performed the Log-rank test to analyze overall survival (OS) based on gene expression.

### Statistical analysis

Cell proliferation, cell migration assays, and xenograft experiments were evaluated using Student’s t-tests and One-way ANOVA with Dunnett’s multiple comparisons test. Statistical assessments were implemented in Graphpad Prism for Mac 8.0 (GraphPad Software, Inc., La Jolla, CA, USA) and JMP 14 software (SAS Institute Japan, Inc., Tokyo, Japan) and p-values of less than 0.05 were considered statistically significant.

## Supplementary Information

Supplementary Information.

## Data Availability

The RNA-seq dataset has previously been reported^19^ and is available on request from the Melbourne Urological Research Alliance (MURAL). ChIP-seq data has been deposited in the Gene Expression Omnibus (GEO) repository (www.ncbi.nlm.nih.gov/geo), accession number GSE193073. The remaining datasets generated during and/or analyzed during the current study are available from the corresponding author on reasonable request.

## Abbreviations

AcH3K27: Acetyl-Histone H3 (Lys27)
ACSL3: Acyl-CoA synthase long-chain family member 3
ADT: Androgen deprivation therapy
ANLN: Aniline-actin binding protein
AR: Androgen receptor
ARBS: AR binding sites
Brn2: Octamer-binding protein 7
ChIP-seq: Chromatin immunoprecipitation sequencing
CRPC: Castration-resistant PC
CTNNB1: Catenin beta 1
DAB: 3,30-Diaminobenzidine
DHT: Dihydrotestosterone
DLGAP5: DLG-associated protein 5
DNPC: AR and NE marker negative PC
ER: Epitope retrieval
FOXA1: Forkhead box A1
GAPDH: Glyceraldehyde-3-phosphate dehydrogenase
GATA2: GATA binding protein 2
GEPIA: Gene expression profiling interactive analysis
IR: Immunoreactivity
MACS: Model-based analysis of ChIP-seq
MTBP: MDM2 binding protein
MURAL: Melbourne urological research alliance
NDRG1: N-Myc downstream regulated 1
NE: Neuroendocrine
NEPC: AR-negative CRPC model with NE characteristics
NRF1: Nuclear respiratory factor 1
OCT1: Octamer transcription factor
OCT4: Octamer-binding transcription factor 4
PC: Prostate cancer
PDX: Patient-derived xenografts
PERP: P53 apoptosis effector related to PMP22
PFN2: Profilin 2
qRT-PCR: Quantitative reverse transcription PCR
RAD21: RAD21 cohesin complex component
RBP1: Retinol binding protein 1
RRID: Research resource identifier
ROSE: Rank ordering of super-enhancers
SAT1: Spermidine/spermine *N*1-acetyltransferase 1
SOX2: SRY-box transcription factor 2
SDC1: Syndecan 1
siSNTB1: SiRNA targeting SNTB1
siPFN2: SiRNA targeting PFN2
SNTB1: Syntrophin beta 1
STR: Short tandem repeat
TCGA: The Cancer Genome Atlas
TFs: Transcription factors
ZIC4: Zic family member 4
