# Supplementary material for: OCT1-target neural gene PFN2 promotes tumor growth in androgen receptor-negative prostate cancer
Source: Sci Rep. 2022 Apr 12;12:6094. doi: 10.1038/s41598-022-10099-x (PMC9005514; doi:10.1038/s41598-022-10099-x)
Supplement: Supplementary file 1 — Supplementary Information. [file 41598_2022_10099_MOESM1_ESM.pdf]

OCT1-Target Neural Gene *PFN2* Promotes Tumor Growth in Androgen Receptor-Negative Prostate Cancer

Daisuke Obinata, Daigo Funakoshi, Kenichi Takayama, Makoto Hara, Birunthi Niranjan, Linda Teng, Mitchell G Lawrence, Renea A Taylor, Gail P Risbridger, Yutaka Suzuki, Satoru Takahashi, Satoshi Inoue

Supplementary Figure S1

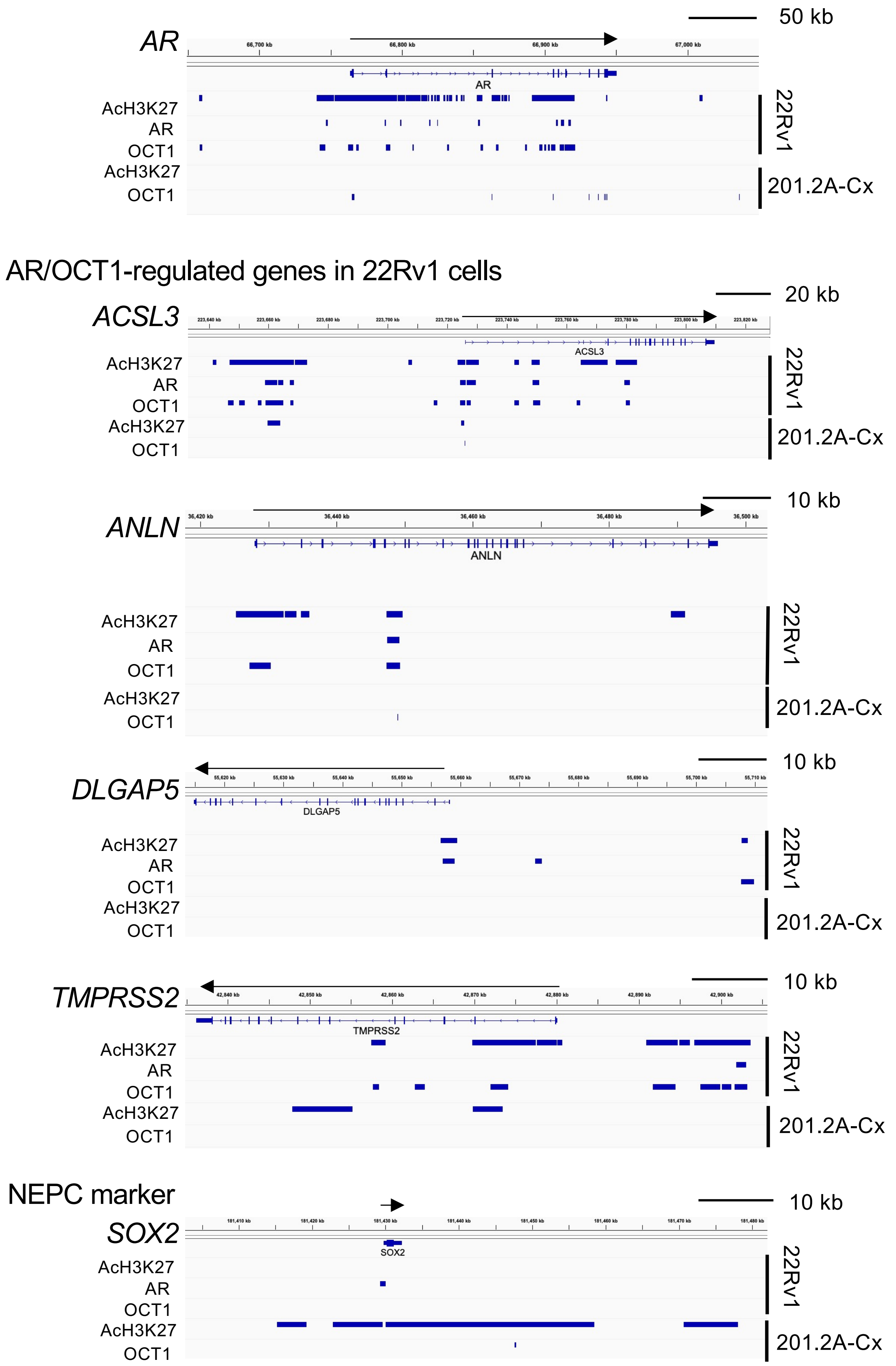

Supplementary Figure S1. ChIP-seq analysis of histone acetylation and OCT1 bindings in *AR*, AR/OCT1-regulated genes and a NEPC marker *SOX2* loci. Significant (*P* < 1E-4) AR/OCT1-binding and active histone modification (AcH3K27) sites in 22Rv1 cells obtained by our past ChIP-seq anlayses (GSE123565, GSE146886) were compared with AcH3K27 and OCT1 binding sites in 201.2A-Cx obtained in this study. *ACSL3*, *ANLN*, *DLGAP5* and *TMPRSS2* were shown as representative AR/OCT1-regulated genes in 22Rv1 cells, AR-positive CRPC model cells. *SOX2* is shown as a NEPC specific marker gene. Arrows indicate the direction of transcription of the gene.

Supplementary Figure S2

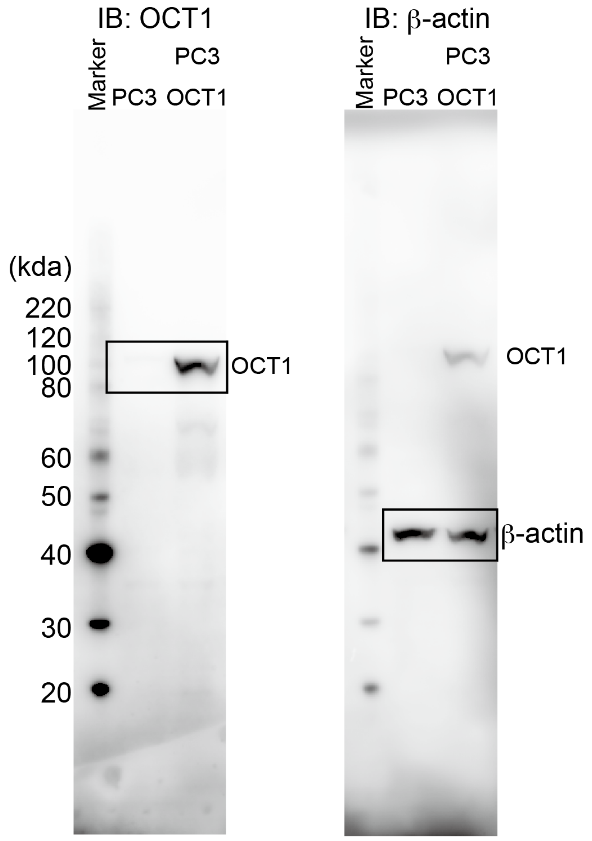

Supplementary Figure S2. Full-length images of Western blotting. The cropped images are shown in Fig. 5A.

Supplementary Figure S3

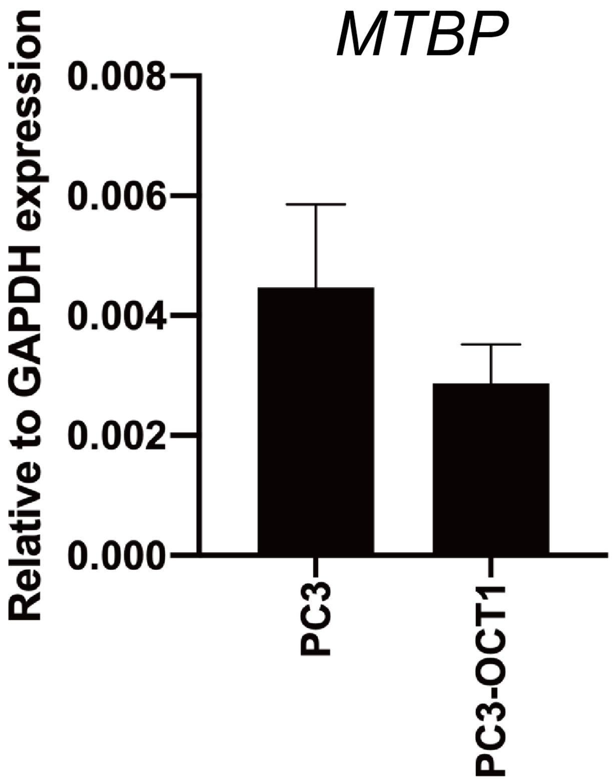

Supplementary Figure S3. Decreased expression of MTBP after OCT1 transient transfection in PC3 cells. Bars, SEM.

Supplementary Figure S4

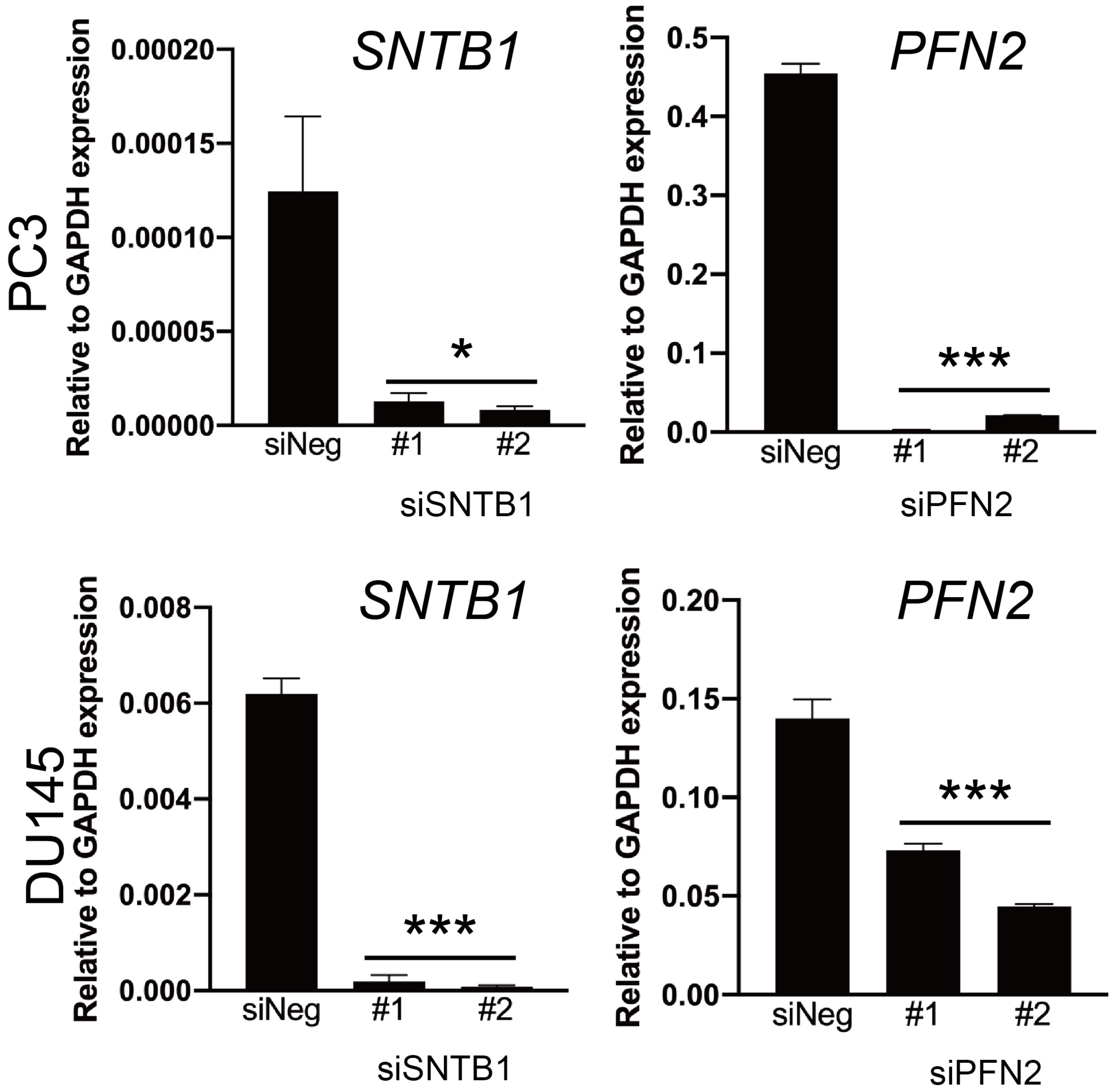

Supplementary Figure S4. The effect of each siRNA on mRNA abundance in PC3 and DU145 cells. Bars, SEM. * P < 0.05, *** p < 0.0001, Student' s t-tests. Bars, SD.

Supplementary Figure S5

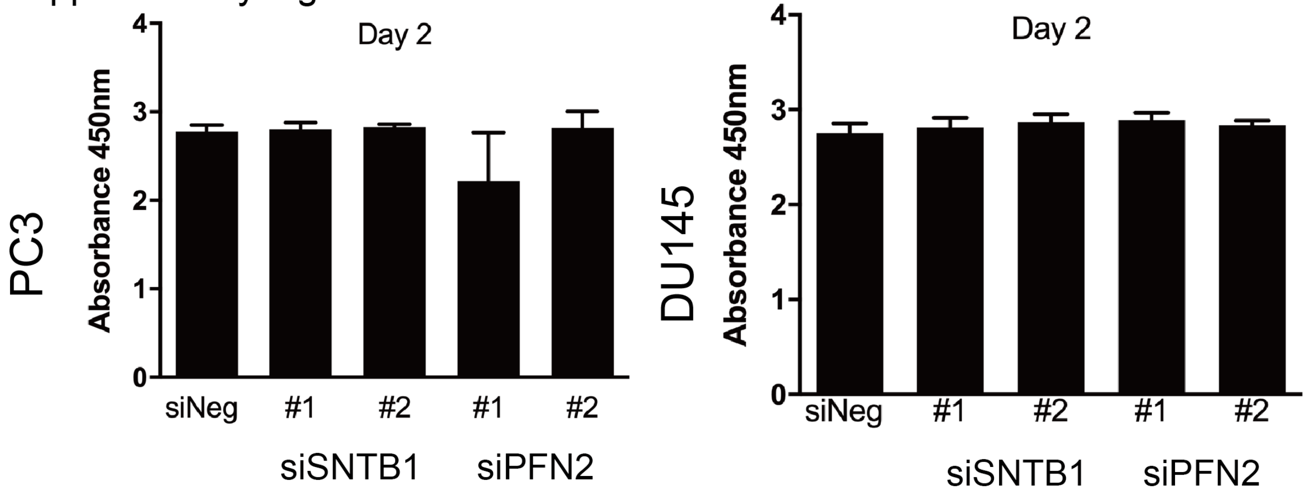

Supplementary Figure S5. The effects of siRNAs on cell proliferation. The effect of siRNA on cell proliferative ability was evaluated using WST-8 on the second day after siRNA treatment. Bars, SD.

Supplementary Figure S6

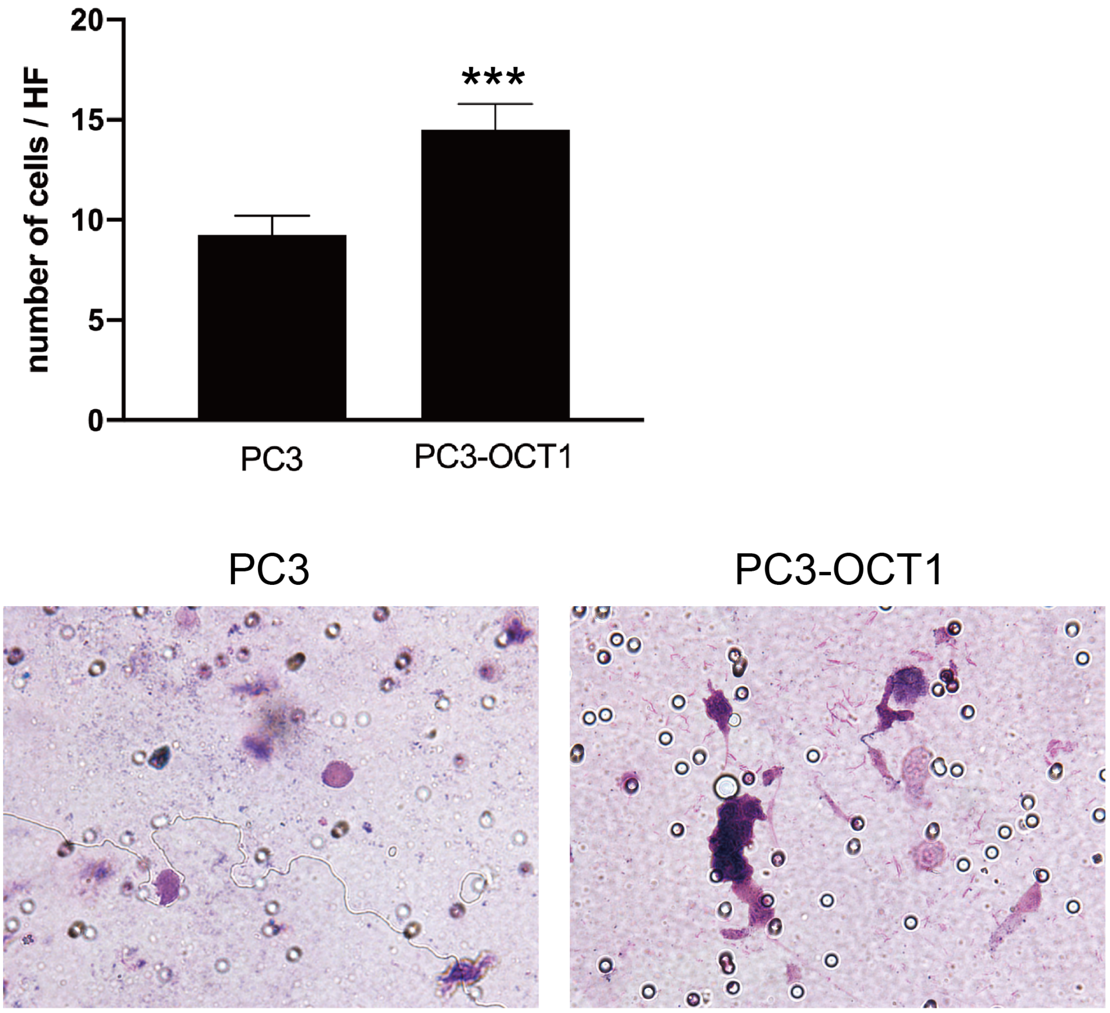

Supplementary Figure S6. Results and representative photographs of cell migration assays with transient overexpression of OCT1. *** $p < 0.0001$, Student' s t-tests. Bars, SD.

Supplementary Figure S7

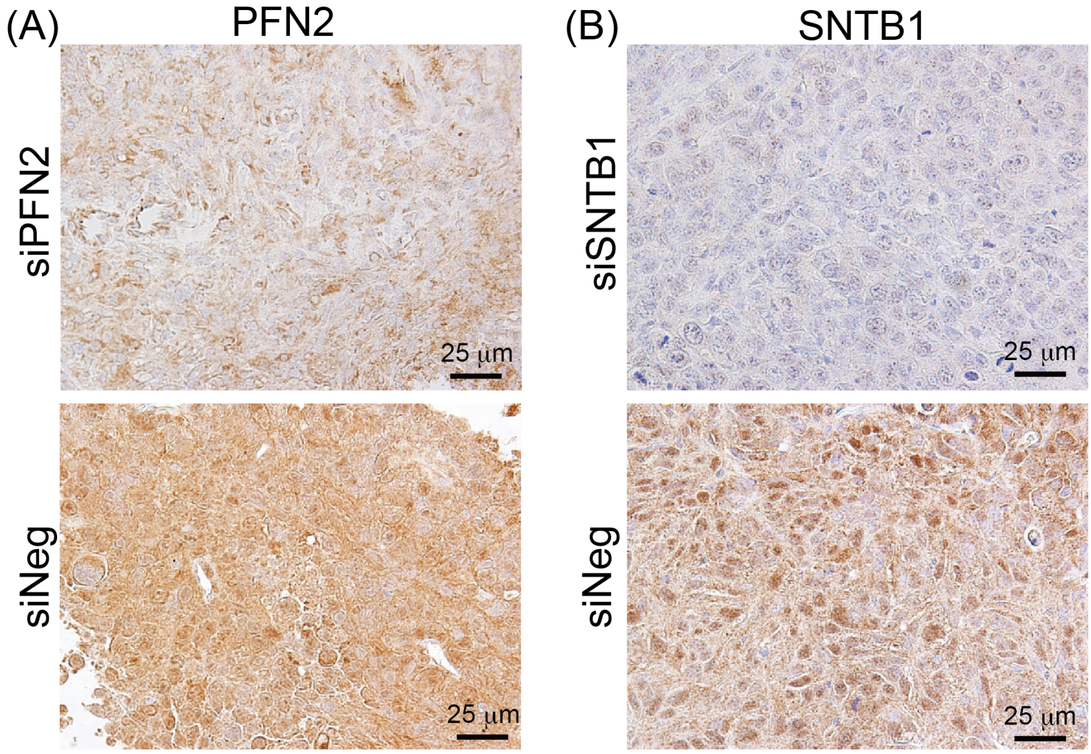

Supplementary Figure S7. Representative immunohistochemistry of PFN2 (A) and SNTB1 (B) in each siRNA-treated PC3 xenograft.

Supplementary Figure S8

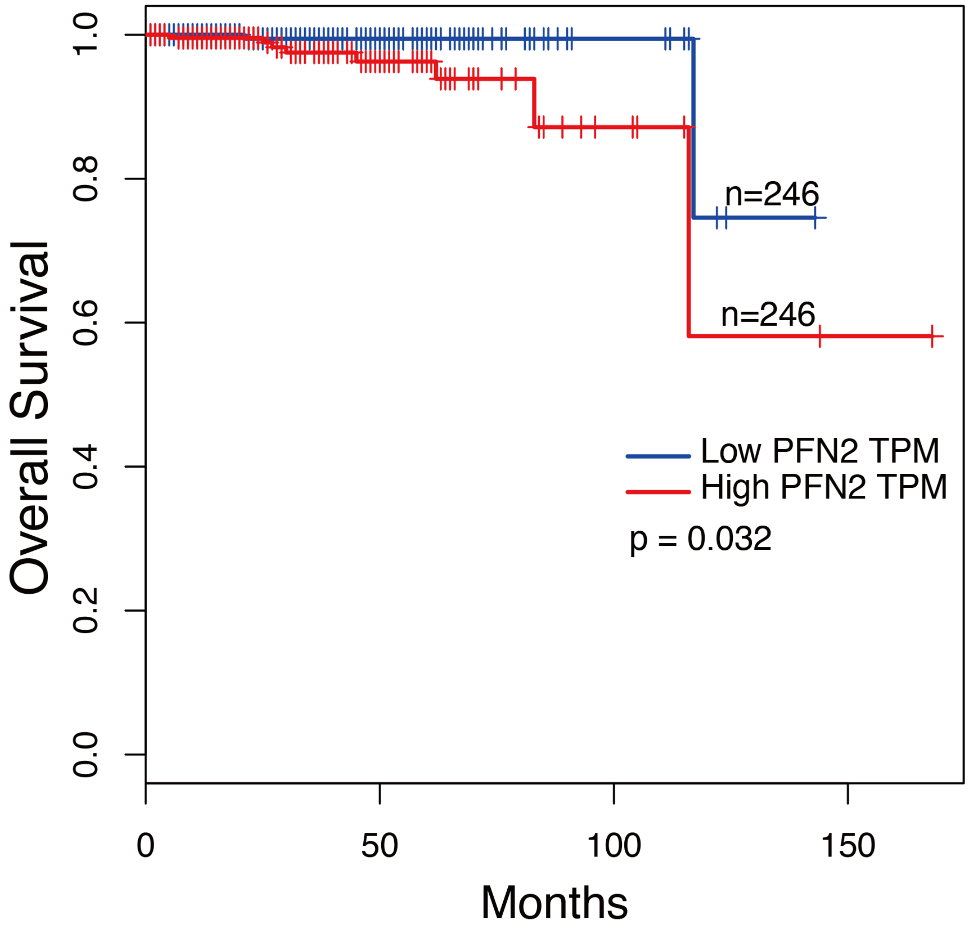

Supplementary Figure S8. High expression levels of PFN2 in prostate tumors are correlated with poor prognosis. We analyzed the overall survival of patients by using GEPIA. The cut off was set at 50 % for high expression and 50 % for low of 492 tumors. P-value was calculated by log rank test (p = 0.032).

Supplemental Table S1. Primers used in qRT-PCR experiments

Gene name	Forward (5′ to 3′)	Reverse (5′ to 3′)
GAPDH	GGTGGTCTCCTCTGACTTCAACA	GTGGTCGTTGAGGGCAATG
SNTB1	GTCCTTTGCAACGCGAACTG	GGTTTTTGTAGGTGCAAGCAGTG
*SNTB1*_MACS_peak_10658	CATGCGGTGGGGTGCATA	TGTAATCTGGTTGTATGGCAGGG
MTBP	ACCGATTCAAAAGGGGGAAAAGA	ATGGAAGAGGGCTCAACACAG
*MTBP*_MACS_peak_10653	GTGGCAGAACTCAAACACTGAA	TGTAATCAAAACGGCTGTGGG
SOX2	GCCCTGCAGTACAACTCCAT	ACATGTGAAGTCTGCTGGGG
*SOX2*_MACS_peak_6774	GCTAATTTCCCCTTTGCGGG	CACGGAGAAAGATGCACCCA
PFN2	CAACGGTTTGACTCTTGGCG	ACACATCAGACCTCCTCAGG
*PFN2*_MACS_peak_6593	TCTGGGTCACTCCTTCTGGG	CTGGGTTGCCTTCCTCCTTC
ZIC4_CHIP	CCCAAGAAAACAAGCTTCCA	AATTGGGCTGGCTAAGGAAT
